# A multipurpose Extracorporeal Life Support Circuit: a concept for multiorgan transplant and circulatory support service Healthcare provider

**DOI:** 10.1051/ject/2023038

**Published:** 2023-12-15

**Authors:** Salman Pervaiz Butt, Nuno Raposo, Yasir Saleem

**Affiliations:** 1 Perfusionist & ECMO Specialist, Cleveland Clinic, Abu Dhabi, United Arab Emirates, Honorary Lecturer University of Bristol, AmSECT Membership No: 8270 PO Box: 112412 United Arab Emirates; 2 Perfusionist, Perfusion Department, Cleveland Clinic Abu Dhabi United Arab Emirates; 3 Clinical Perfusionist, Department of CTVS, All India Institute of Medical Science-Rishikesh

**Keywords:** Extracorporeal life support, Versatile circuit, ECMO, VAD, VV bypass, Versatility in applications

## Abstract

The demand for efficient and adaptable life support systems in the field of Extracorporeal Life Support (ECLS) is steadily increasing. To meet this growing need, there is a requirement for a versatile extracorporeal life support circuit that can be effectively applied in various medical scenarios, especially in tertiary hospitals where multiple ECLS services are utilized. These services include Extracorporeal Membrane Oxygenation (ECMO) for addressing respiratory or cardiac problems, Ventricular Assist Device (VAD) as a bridge to recovery or heart transplant, and Venovenous Bypass (VVB) for assisting liver transplantation. In light of this, we propose the creation of a multipurpose circuit that integrates multiple extracorporeal life support (ECLS) functions to cater to diverse medical needs. This innovative circuit not only offers cost-effectiveness and enhanced safety but also ensures optimal utilization, thereby revolutionizing the realm of life support technologies.

## Design

ECLS applications demand tailored configurations and circuit modifications to address individual patient requirements, which can include additional cannulas or changing cannulation sites. Additionally, specialized procedures like lung transplantation may necessitate a hybrid ECMO and cardiopulmonary bypass (CPB) circuit to ensure optimal patient care [1–3].

We propose a design for a versatile ECLS circuit suitable for various clinical scenarios. Our objective was to create a customized ECLS system that could easily be manufactured by any of the existing market manufacturers and could be employed in different ECLS modes. Currently, we utilize ECLS in the forms of ECMO (both VV and VA configurations), VAD, and VVB for liver transplantation and they all come as separate consumables and require separate machines.

**Figure 1:**
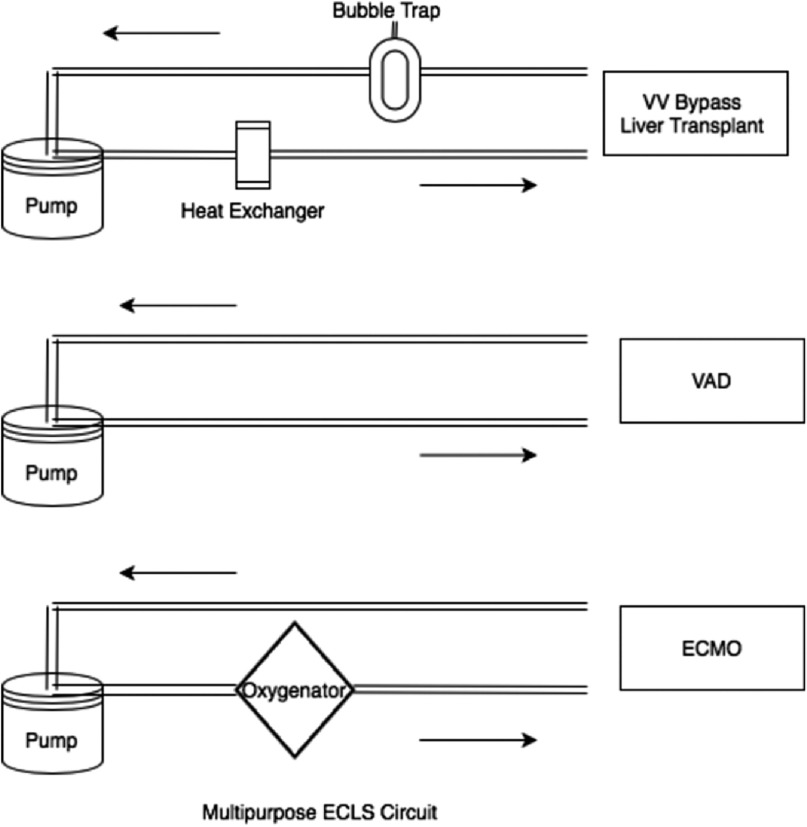
Illustration of Multipurpose ECLS Circuit.

To accomplish this, the circuit base configuration consists of three essential components: a centrifugal pump, a venous bubble trap (VBT), and a heat exchanger (HE) (refer to Figure 1 for configurations). The bubble trap and the heat exchanger have connections before and after each of these devices to facilitate the alteration of the ECMO or VAD circuit.

For instance, if the base circuit requires converting to VAD, both VBT and HE can be excluded. In the case of converting the base circuit to an ECMO circuit, VBT can be excluded and HE can be replaced with an ECMO oxygenator.

**Figure 2:**
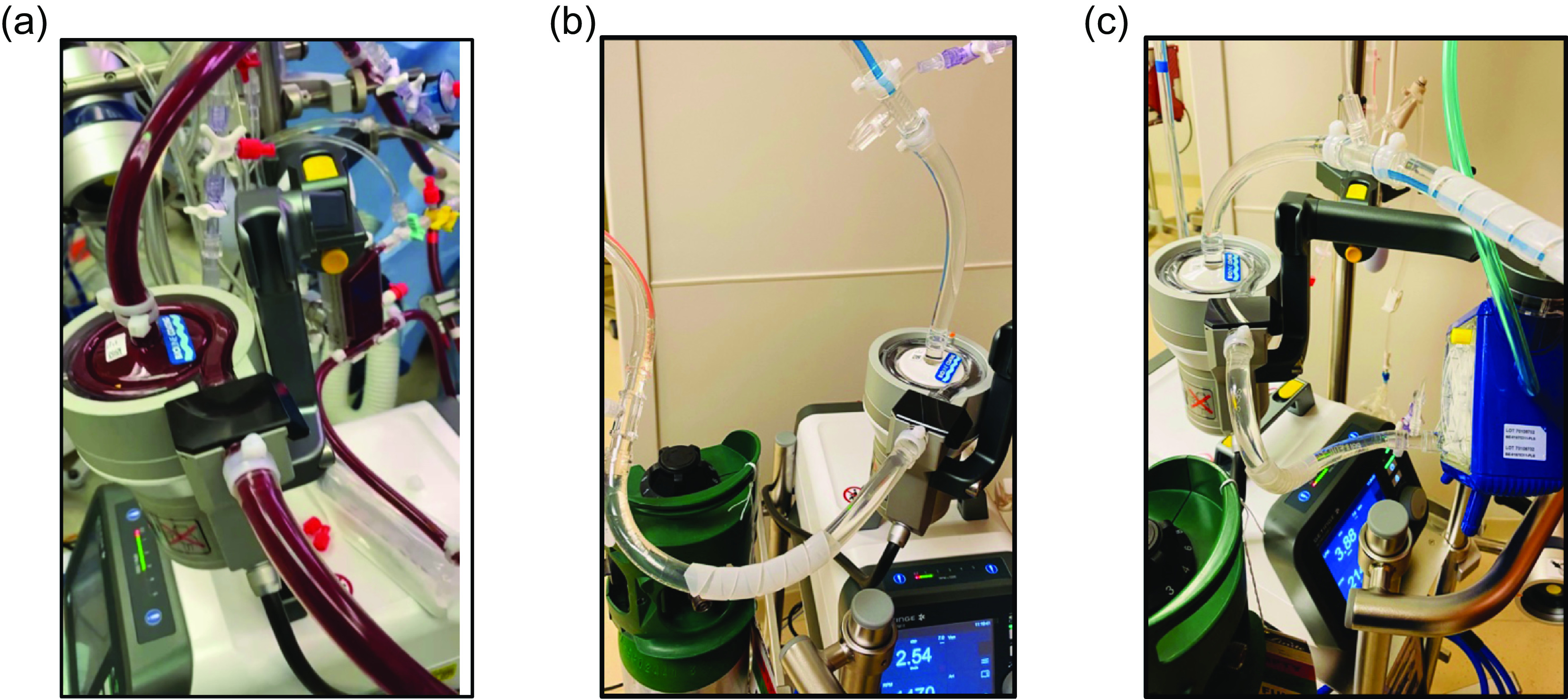
Illustration of Multipurpose ECLS Applications (a) Veno-Veno Bypass; (b) Ventricular Assist Device; (c): ECMO.

From a hardware standpoint, the RotaFlow II and Maquet’s Heater Cooler are utilized for these applications. In terms of consumables, Maquet’s centrifugal pump (MCP) and Sorin cardioplegia device serve as heat exchangers for VV Bypass, the MCP in conjunction with its circuit is employed for VAD applications, and MCP, oxygenator with circuit are employed for ECMO applications (Figure 2).

The multipurpose extracorporeal life support circuit provides a wide range of benefits, including cost-effectiveness, safety, patient care, workflow efficiency, training, and resource optimization.

From a cost-effectiveness perspective, this circuit consolidates four applications into a single system, optimizing equipment utilization and eliminating the need for separate circuits for each procedure. This consolidation reduces costs by maximizing efficiency and eliminates the expenses associated with maintaining and operating multiple devices.

The concept of a primed circuit significantly enhances both cost-effectiveness and safety. Unlike traditional circuits that may remain idle for extended periods, the multipurpose circuit is always primed and ready for immediate use. This proactive approach prevents wastage of resources that would occur if the circuit were unused and ensures that the system is readily available whenever needed. Additionally, the circuit’s adaptability to different scenarios ensures efficient resource utilization, preventing it from becoming obsolete.

The advantages of a primed circuit extend beyond cost-effectiveness. Its immediate readiness minimizes delays in initiating life support, allowing for prompt and timely intervention when required. This feature enhances patient care by ensuring quick access to life support without unnecessary complications or waiting times.

The multipurpose circuit also simplifies training requirements for healthcare professionals. With only one system to familiarize themselves with, medical teams can easily learn and master its operation. This promotes standardized training protocols, facilitates knowledge sharing among team members, and reduces the learning curve associated with using multiple devices.

Furthermore, the circuit contributes to resource optimization by reducing the number of devices required. By consolidating multiple functions into a single system, it helps hospitals optimize storage space, minimize equipment maintenance, and improve inventory management. This streamlined approach creates a more organized and efficient healthcare environment.

In summary, the multipurpose extracorporeal life support circuit offers cost-effectiveness, safety, enhanced patient care, streamlined workflow, simplified training, and resource optimization. These benefits positively impact both healthcare professionals and the patients they serve, making it a valuable tool in the medical field.

## Limitation

The implementation of a multipurpose circuit would yield significant advantages in healthcare settings where an ECLS circuit is needed for diverse specialties. Additionally, it would prove particularly beneficial in markets facing financial constraints.

## Conclusion

In conclusion, the development of a versatile extracorporeal life support (ECLS) circuit offers a practical and achievable concept. Combining multiple functions into a single system optimizes equipment utilization, reduces costs, and enhances patient care. This streamlined approach simplifies training, improves workflow efficiency, and optimizes resource management. The multipurpose ECLS circuit is a feasible solution that can revolutionize life support technologies and benefit both healthcare professionals and patients.
